# Epicystotomy

**Published:** 1858-06

**Authors:** 


[The following is part of an article by E. Noeggerath, M. D., of New York, on Epicystotomy. The first part of the paper details a case of stone, in which the calculus was removed by the knife operation.]

Every surgeon who is called upon to treat a patient for stone in the bladder, will prefer the operation with the knife or the lithotrite to the tedious application of internal remedies for dissolving the stone. This will be generally admitted. On the other hand, every physician who has treated cases of concretions in the bladder, must have felt a desire of having a preparation, which might enable him to dissolve stone. There are patients who will rather bear their sufferings than be approached with an instrument, or if once operated upon, decline any further surgical interference. Again, we often meet with such a high state of irritability of the bladder, that it would be unsafe to increase it by a protracted employment of steel instruments. In other cases the calculus is small, so that we do not feel justified to subject the patient to all the hazards of a regular lithotomy for relieving him from the trifling uneasiness connected with his disease, especially if the condition of the urethra or the location of the stone is such that we must abstain from lithotrity. This is not all. We meet with hundreds of unforseen circumstances, which make it desirable to have a solvent for stone.

Various attempts have been made to dissolve stone in the bladder, sometimes by introducing remedies into the system by the digestive organs, sometimes by direct application to the calculus. It is the latter ones that we intend to subject to a short analysis, to see if any of them is worthy of attention. Until within the last few years, the therapeutical treatment of stone has been the almost exclusive privilege of irregular practitioners. This is one reason that it is but recently that some really scientific men have taken the matter in their own hands. The remedies used by the latter may be divided into two classes, viz.: 1, simple solvents; 2, chemical agents, working on the principle of double decomposition. Among the former are to be counted, (*a*) acids (for phosphatic stones,) such as muriatic acid, nitric acid (Brodie,) gastric juice (Dorsey, Millot;) (*b*) alkalies (for lithic calculi,) lime water (Berzelius, Amussat, Leroy,) borax (Ure, Gay Lussac,) Vichy water (Petit, Chevallier,) Baréges water (Desault.) It would surpass the limits proposed for this article, if we were to discuss the value of these remedies. I shall, therefore, confine my remarks to those of the second class, viz., agents acting upon the stone by double decomposition.

The chemicals proposed for this purpose are preparations of lead, and among these the nitro-saccharate of lead has been applied in practice. Some of our greatest surgeons, and among them Prof. Gross, of Philadelphia, when speaking of lead preparations as solvents for stone, show clearly that they have not the least confidence in their application. But the observations of Dr. Hoskins, of Guernsey, England, made such a favorable impression upon my mind that I was induced to make some further inquiries regarding the solubility of stone by the method of double decomposition.

Instead of nitrate of lead I employed the acetate (plumbum aceticum,) thinking that the presence of an organic acid would have a greater affinity for a chemical compound formed in a living organism. Before using it as an injection, I instituted the following experiments. In one ounce of water I dissolved 10 grains of acetate of lead, and superacidulated it with 10 drops of strong acetic acid. This clear solution I poured over ten grains of stony particles taken from the outer layer of the calculus. As soon as they came in contact with the water thus saturated, it seemed as if whitish vapors began to settle around each of them, emitting at once, small air-bubbles. These clouds grew thicker and thicker, until at last the particles were entirely enveloped. I now began to agitate the fluid by moving the bottle slowly up and down for about half an hour, the consequence of which was, that the formerly clear solution turned quite thick, thus proving that a chemical process was going on. The mixture, when examined the next day, presented the following appearance. The fluid portion was almost clear, while the bottom of the vial was covered with a thick, coherent precipitate, which consisted of three portions; (*a*) some few of the stony articles left coherent, but all greatly diminished in size; (*b*) a greyish, powdery concretion, being seemingly a mixture of disintegrated particles of the stone, and precipitated portions of the lead; (*c*) a layer of a perfectly white substance shaped in a coherent thin foil. After filtering the liquid from the solid portion, I separated the three different specimens of residue as much as possible. I was able to collect six of the fifteen small pieces of stone which I had subjected to the test. While in bulk, they formed about the third part of the original stock, they had lost so much of their weight, that they weighed not more than *one grain*, and were so much changed in their constitution that the slightest touch with the finger was sufficient to crush them into powder. Another portion of the calculus was reduced to a very thin dust.

The white layer (*c*) I collected on charcoal, and exposed it to the blowpipe, when to my great surprise, it melted away to a greenish white bead, which after cooling crystallized so evidently that not a shade of doubt could be left about its nature—it was *phosphate of lead.*

The liquid portion filtered from the solid mass exhibited a strong acid reäction to test paper. A stream of hydrosulphuric acid, being transmitted through the fluid effected not the least change in it; thus proving that the ten grains of acetate of lead taken for the experiment, had been entirely reduced. A further chemical examination showed that the liquid contained a great quantity of lime, and some ammonia, in solution, the acids combined with them, being phosphoric and acetic acid, the latter in excess.

The conclusions drawn from this experiment may be expressed as follows: A superacidulated solution of acetate of lead acts upon phosphatic stones as a decomposing, disintegrating, and solvent agent; the phosphoric acid previously combined with lime or ammonia, uniting with the lead, the acetic acid of which goes over to a portion of the lime or ammonia, in exchange for phosphoric or carbonic acid, while the free acetic acid in the water renders a portion of the undecomposed phosphate of lime soluble.

Further, it became evident that ten grains of the lead are not sufficient to decompose ten grains of a phosphatic stone. But this has no value in practice. The particles of stone which were left unchanged in size and shape had undergone such changes that agitation in water sufficed to turn them into powder.

This one experiment speaks clearly enough, and not the least shade of doubt can be left that acetate of lead is a remedy by which we are enabled to destroy and dissolve phosphatic stones. I therefore resorted to it with confidence in its efficacy, and I was not deceived. The remaining particles of stone in the bladder and prostate were dissolved. In short it acted admirably in the manner stated above. Its employment was attended with no difficulty at all; it produced neither pain nor irritation in the urethra or bladder, and the happy result of our operation is due, to a certain extent, to these lead injections.

The formula I used was as follows:

℞ Plumbi acetic. grs. vi.
S. in aqua. fontan. ℥vi.
Adde acidi acetic. fort. q. s.
ad solution. perfect.

D. S.—One small syringeful to be injected twice a day.—*New York Journal of Medicine.*

